# Advanced glycosylation end products inhibit the proliferation of bone-marrow stromal cells through activating MAPK pathway

**DOI:** 10.1186/s40001-021-00559-x

**Published:** 2021-08-18

**Authors:** Zheng Li, Xiao Wang, Tian-pei Hong, Hao-jie Wang, Zhan-yi Gao, Meng Wan

**Affiliations:** 1 Department of Stomatology, Peking University Third Hospital, No. 49 Huayuan North Road, Haidian District, Beijing, 100191 China; 2 Department of Endocrinology, Peking University Third Hospital, Beijing, 100191 China

**Keywords:** Advanced glycosylation end products, MAPK pathway, Bone-marrow stromal cells, Proliferation

## Abstract

**Background:**

The purpose of present study was to explore the mechanism of nuclear factor-kappa B (NF-κB), phosphatidylinositol 3-kinase (PI3K)/protein kinase B(PKB/Akt) and mitogen-activated protein kinase (MAPK) signaling pathways after intervention of advanced glycosylation end products (AGEs) on rat bone-marrow stromal cells (BMSCs).

**Methods:**

Prepare and identify AGEs. BMSCs were isolated from 16 SD rats and cultured with different concentration of AGEs. Cell viability was detected by cell counting kit-8 (CCK-8). BMSCs were cultured with AGEs (0.25 mg/ml) for 30 min, 12 h, 24 h, 72 h and 120 h. In addition, BMSCs were cultured with AGEs, AGEs + JNK inhibitor and AGEs + P38 inhibitor for 24 h and 48 h, respectively. Western blotting and RT-PCR were used to determine the protein and mRNA expression levels, respectively.

**Results:**

Cell viability of BMSCs was significantly correlated with concentration and effect time of AGEs (*P* < 0.05), and the most appropriate concentration was 0.25 mg/ml. AGEs stimulation significantly increased the protein expression levels of NF-κB p65, JNK, p38 (*P* < 0.05), decreased IκB (*P* < 0.05), but had no effect on the protein expression of Akt in BMSCs (*P* > 0.05). At the mRNA level, JNK and p38 inhibitors significantly reduced the levels of NF-κB p65, p38 and JNK, increased IκB (*P* > 0.05), but had no effect on Akt in BMSCs (*P* > 0.05). At the protein level, JNK and p38 inhibitors notably decreased the expression of NF-κB p65, p38, p-JNK, P-IκB and JNK (*P* < 0.001), and increased IκB (*P* < 0.05).

**Conclusion:**

Advanced glycosylation end products can inhibit the proliferation of bone-marrow stromal cells through activating MAPK pathway.

## Background

Diabetes mellitus and periodontal disease are chronic diseases related to lifestyle with high prevalence worldwide [1]. Epidemiological studies show that diabetes can affect periodontal health [2]. The biological pathogenesis involved is related to the cell and molecular level regulation of hyperglycemia on periodontal tissue [3–5]. In 2013, the European Periodontal Association (EFP) and the American Periodontal Association (AAP) jointly proposed the possible correlation mechanism between diabetes and periodontitis [6].

Under the condition of hyperglycemia, advanced glycosylation end products (AGEs) and its main signal receptor RAGE interact with each other, which can lead to the abnormal function of immune cells, change the phenotype and function of other important cells in periodontal tissues, and increase the expression of pro-inflammatory cytokines (Interleukin (IL)-6, IL-1β, tumor necrosis factor (TNF)-α) [7]. At the same time, hyperglycemia increases the over expression of reactive oxygen species (ROS) and causes oxidative stress [8]. It can directly or indirectly change the quantity and quality of various cytokines through the AGEs/RAGE response axis. In addition, under the condition of hyperglycemia, the proportion regulation of RANKL/OPG can also lead to inflammation and destruction of periodontal tissue [9]. The change of biomembrane ecosystem of subgingival plaque and the increase of adipocytokine secretion caused by diabetes related obesity and dyslipidemia play a synergistic role, ultimately leading to the loss of metabolism balance of periodontal tissue, which is manifested in the increase of periodontal tissue damage and tissue repair function damage, that is, rapid progress and serious periodontitis [10].

Osteoclasts and osteoblasts are required to participate in the reconstruction of alveolar bone in periodontitis [11]. Therefore, it is important to study the molecular mechanism of alveolar bone destruction, absorption, reconstruction and repair in hyperglycemia to elucidate the biological cause of chronic periodontitis caused by diabetes [12]. AGEs is a non-enzymatic glycosylation product of protein, including collagen and other components, under the condition of chronic hyperglycemia. As a characteristic expression of diabetes, it plays a key role in the pathophysiological process induced by hyperglycemia. Therefore, the purpose of present study was to explore the effect of AGEs on bone-marrow stromal cells (BMSCs) in rats.

## Materials and methods

### Chemicals and antibodies

Bovine serum albumin (BSA) was obtained from Shanghai Hengyuan Technology Biology Co., Ltd (Shanghai, China). Glucose, Phenylmethylsulfonyl Fluoride (PMSF), and ethylene diamine tetraacetic acid disodium salt (EDTA) were obtained from Beijing solabo Technology Co., Ltd. (Beijing, China). Penicillin and streptomycin were bought from Sangon Biotech Inc. (Shanghai, China). Ethanol (purity > 99%) was obtained from Xilong Chemical Co., Ltd. (Guangdong, China). Fetal bovine serum (FBS) was bought from Sangon Biotech, Inc. (Shanghai, China) and Dulbecco’s modified eagle medium (DMEM) was purchased from ThermoFisher (MA, USA). CCK-8 kit was obtained from Shanghai Yanjin Biotechnology Co., Ltd. (Shanghai, China). Anti-p-P38, anti-P38, anti-JNK, anti-p-JNK, anti-NF-κB p65, anti-p-NF-κB p65, anti-IκB, anti-p-IκB, anti-AKT and anti-p-AKT antibodies were purchased from Abcam Technology (Cambridge, UK). Anti β-actin antibody, Goat anti-rabbit and mouse IgG and mouse anti-goat secondary antibodies were purchased from ZSGB Biotech Co., Ltd. (Beijing, China). Unless specified, all other reagents are obtained from Sigma Chemical Co. (St. Louis, MO, USA).

### AGE’s preparation

2.5 g BSA (50 mg/ml), 4.045 g D-glucose (500 mmol/l), 0.003 g penicillin (100 IU/ml), 0.005 g streptomycin (100 IU/ml), 0.0130642 g benzenesulfonylfluoro (1.5 mmol/l) and 0.009306 g disodium EDTA (0.5 mmol/l) were dissolved in 50 ml PBS solution with 0.2 mmol/l and pH 7.4). After the reagent was fully dissolved, it was filtered by 0.22 μM filter membrane and put into sterile test tube. PBS incubated with BSA but without D-glucose was prepared as control under the same conditions. The prepared AGEs were placed in a sterile tube, sealed with a sealing film, and then placed in an incubator at 37 °C for 90 days to obtain brown products. After incubation, d-glucose which was not combined with BSA was removed by PBS dialysis for 48 h, and then filtered by 0.22 μM filter membrane. According to the specific fluorescence characteristics of AGEs, that is, the fluorescence of 440 nm emission wavelength can be produced under the excitation light of 370 nm wavelength, the fluorescence values of incubated AGEs–BSA and the control were determined by FLX-800 fluorescence spectrophotometer. Finally, put the prepared ages into the refrigerator at − 20 °C until use.

### Isolation and culture of bone-marrow stromal cells (BMSCs)

The animal experiments were approved by the Institutional Ethical Committee of Peking University Third Hospital. A total of 16 Sprague–Dawley (SD) male rats, aged 4 months, were purchased from the Experimental Animal Centre of Peking University Third Hospital and maintained under standard living conditions (room temperature of 22 ± 2 °C, 50–60% relative humidity, and 12 h dark/light cycle). One week after adaptive feeding, SD rats were anesthetized with 50 mg/kg pentobarbital sodium and soaked in 75% ethanol for 5 min. The femur was separated under aseptic condition, the metaphysis was cut off, and the marrow cavity was washed several times with culture medium. The cell suspension was transferred into the test tube and filtered with 100 mesh and 200 mesh stainless steel filters to obtain the single cell suspension. Two drops of red blood cell (RBC) lysate was added and the cells were collected by centrifugation at 1000 rmp/min for 10 min. DMEM medium containing 15% FBS was used to cultured cells. The cells were adjusted to 10^7^ cells/ml, and cultured in 37 °C and 5% CO_2_ incubator. When the suspension cells were removed, the adherent cells were BMSCs. L-02 cells were maintained in a humidified incubator with a 5% CO_2_/95% air atmosphere at 37 °C and passaged daily by trypsin–EDTA digestion (Solarbio Co., Beijing, China).

### Cell counting kit-8 (CCK-8) assay

BMSCs in logarithmic growth phase inoculated in 96-well plate (5 × 10^5^ per well). After attachment to the plates, BMSCs were cultured with 0.125, 0.25, 0.5, 1.0 and 2.0 mM AGEs. At 24 h, 48 h and 72 h, 10 µl CCK-8 reagent was added to each well, and the cells were continued to culture for 1 h. The optical density (OD) was detected at 490 nm by a microplate reader (Bio-Rad, Inc., Hercules, CA, USA), and the IC50 value was calculated.

### Grouping

BMSCs in logarithmic growth phase inoculated in culture flask with 25 cm^2^. To explore the mechanism of AGEs in the proliferation of BMSCs, BMSCs were divided into 12 groups. Cells were cultured with PBS or 0.25 mM for 30 min, 12 h, 1 day, 3 days and 5 days, respectively. Furthermore, BMSCs were divided into 12 groups. Cells were cultured with PBS, 0.25 mM AGEs, JNK inhibitor (SP600125), 0.25 mM AGEs + JNK inhibitor, P38 inhibitor (SB203580), 0.25 mM AGEs + P38 inhibitor for 24 h or 48 h. After incubation, cells in each group were collected for subsequent experiments.

### RT-PCR

Total RNA from BMSCs was obtained using a total RNA isolation kit (Tiangen Biotech Co., Ltd., Beijing, China) according to the directions. Total RNA was reversibly transcribed to cDNA using cDNA synthesis kit (TIANScript cDNA; Tiangen Biotech) according to the specification. RT-PCR was carried out by MJ PTC-200 PCR system (Bio-Rad) and RT-PCR kit (Aidlab Biotech Co., Ltd., Beijing, China). Specific primers (Table 1) are shown in Table 1. mRNA was measured by real-time PCR. Relative gene expression levels were calculated using the 2^−∆∆^Ct method (with β-actin used as the reference gene) and normalized as indicated.

**Table 1:** Primer sequences

Genes	Forward primer (5′–3′)	Reverse primer (5′–3′)	length (bp)
NF-κBp65	CGGATTGAAGAAAAACG	TTGAAAAGGCATAGGGC	169 bp
JNK	TTAGATGAAAGGGAGCA	TGACGCCATTCTTAGTT	91 bp
P38	CAGATGCCGAAGATGAAC	CCTCTTATCCGAGTCCAA	99 bp
Akt	CACAGGTCGCTACTATGCC	GGTCGTGGGTCTGGAATG	152 bp
IκB	CTGCGTGATGAAGATTGGA	GCATCCCTGAGCAGTTGG	116 bp
β-actin	GGCACCACACCTTCTAC	CTGGGTCATCTTTTCAC	107 bp

### Western blotting

The total protein of BMSCs were homogenized in cold RIPA buffer (Beyotime Institute of Biotechnology, Shanghai, China). Supernatants were collected after centrifuging at 12,000×*g* for 20 min at 4 °C. Protein concentration was measured using a BCA protein concentration assay kit (Beyotime Institute of Biotechnology, Shanghai, China). Protein were separated by SDS-PAGE and transferred to nitrocellulose membrane. The membrane was blocked for 1 h at room temperature with nonfat dried milk or BSA. Membranes were incubated with primary antibodies overnight at 4 °C, followed by incubating secondary antibodies for 1 h at room temperature. Then, they were exposed to ECL Western Blotting Substract reagent (Tsea biotech Co., Shanghai, China). Molecular Imager Chemi Doc XRS (BIO-RAD Co., California, USA) and JS-780 automatic gel imaging analysis systems were used for blotting and quantitative analysis. β-actin was used as the internal control.

### Statistical analysis

SPSS22.0 software (International Business Machines, corp., Armonk, NY, USA) was used for statistical analysis. The measurement data were expressed as means ± standard deviation (SD) and tested for normal distribution. The difference between groups was compared by independent sample *t* test. One-way analysis of variance (ANOVA) was used to compare the difference among the groups. The data of repeated measurement were analyzed by general linear model (GLM). *P* < 0.05 was considered statistically significant.

## Results

### Preparation of AGEs and rat BMSCs

**Fig. 1:**
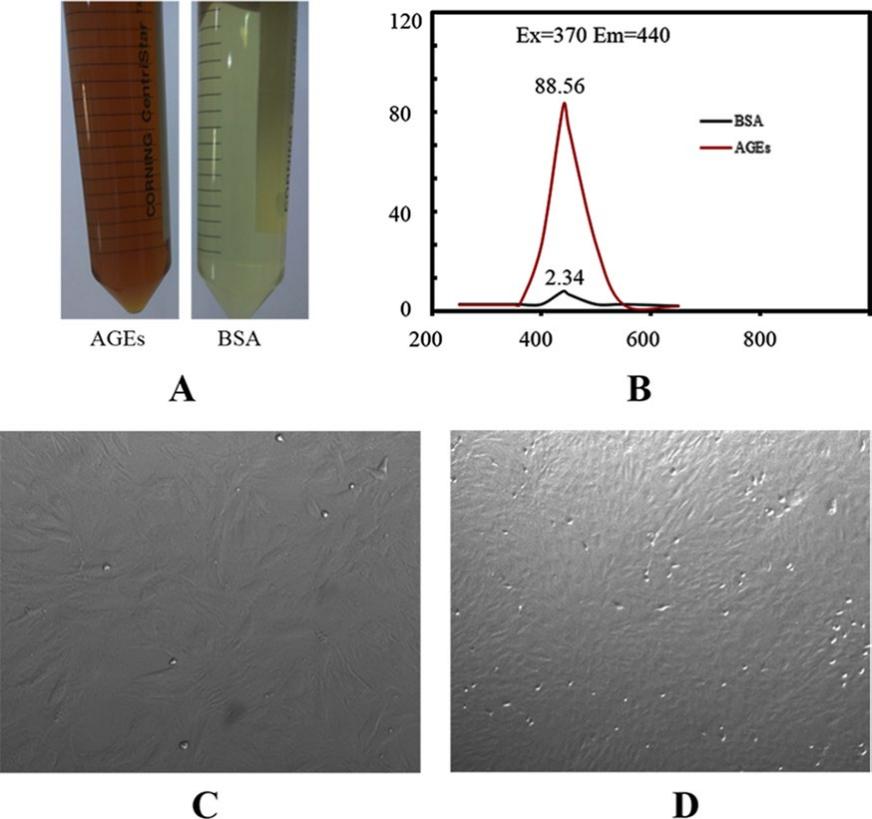
AGEs and rat BMSCs. **A** AGEs and BSA. **B** Fluorescence intensity. **C** BMSCs of primary generation. **D** BMSCs of third generation. Images (100× magnification) are representative images of three independent samples

The fluorescence intensity of AGEs reagent was 88.56 U/mg and that of BSA was 2.34 U/mg **(**Fig. 1A, B**)**. The results showed that it was consistent with the spontaneous fluorescence characteristics of AGEs. Furthermore, as shown in Fig. 1C, D, BMSCs were scattered at the bottom of the dish. Some cells adhered to the wall with different sizes and oval clusters. With the increase of time, the cells became long fusiform, many colonies were formed and arranged radially.

### Effect of AGEs on cell viability of BMSCs

**Fig. 2:**
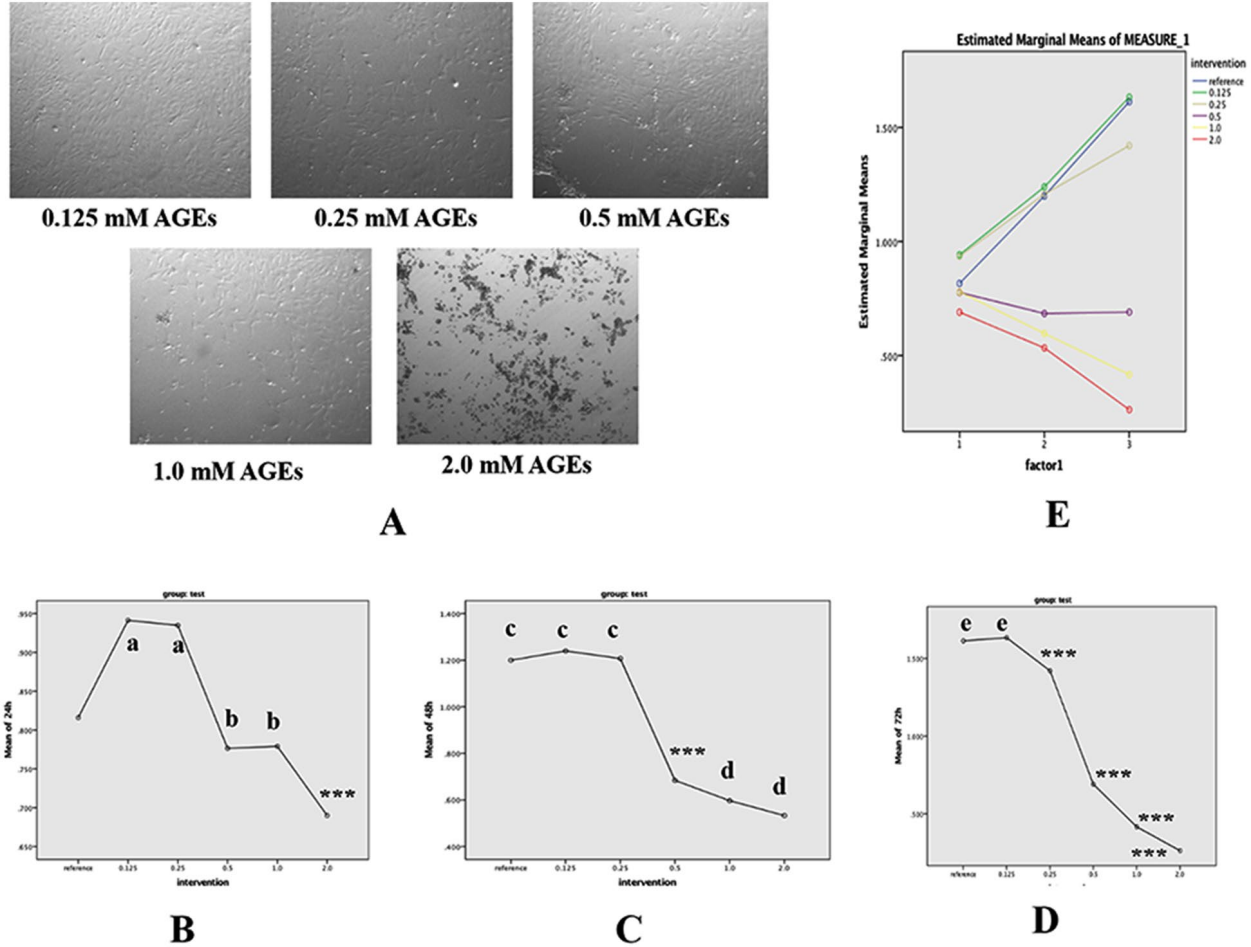
Effect of AGEs on cell viability of BMSCs. **A** Effects of different concentrations of AGEs on the differentiation of BMSCs. **B** Effects of different concentrations of AGEs on the differentiation of BMSCs for 24 h. **C** Effects of different concentrations of AGEs on the differentiation of BMSCs for 48 h. **D** Effects of different concentrations of AGEs on the differentiation of BMSCs for 72 h. **E** Effects of action time and concentration of AGEs on cell vitalities of BMSCs. Images (100× magnification) are representative images of three independent samples. Data are expressed as means ± SD. ***P < 0.001; ^a, b, c, d, e^*P* < 0.001 vs. control group (ANOVA, *F* = 20.8–157.7)

BMSCs under the effect of AGEs at different concentrations after 48 h of culture are shown in Fig. 2A. It suggested that the proliferation of the cells was significantly inhibited by AGEs, and the higher the concentration of AGEs, the more obvious the inhibition. The effects of different AGEs (2, 1, 0.5, 0.25 and 0.125 mM) on proliferation of BMSCs in 24, 48 and 72 h are shown in Fig. 2B, C and D. At each time, the cell viability was decreased with the increase concentration of AGEs with significant differences between the groups (*P* < 0.05). Besides, GLM analysis showed that the cell viability was significantly correlated with concentration, time and their interaction of AGEs (*P* < 0.05), and when the concentration was more than 0.5 mM, the cell viability was significantly decreased with effect time (Fig. 2E).

### Effect of AGEs on MAPK signaling pathway

**Fig. 3:**
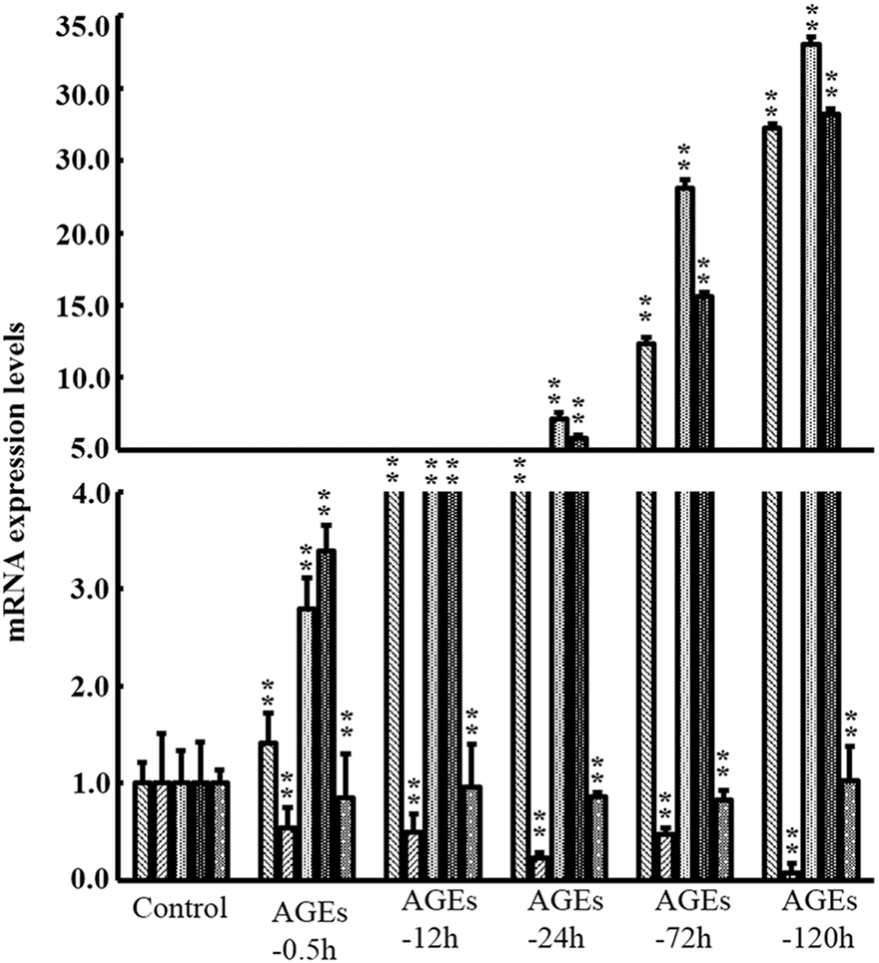
Effect of AGEs on mRNA expression levels of factors in MAPK signaling pathway. Data are expressed as means ± SD (*n* = 3). ***P < 0.001 vs. control group

**Fig. 4:**
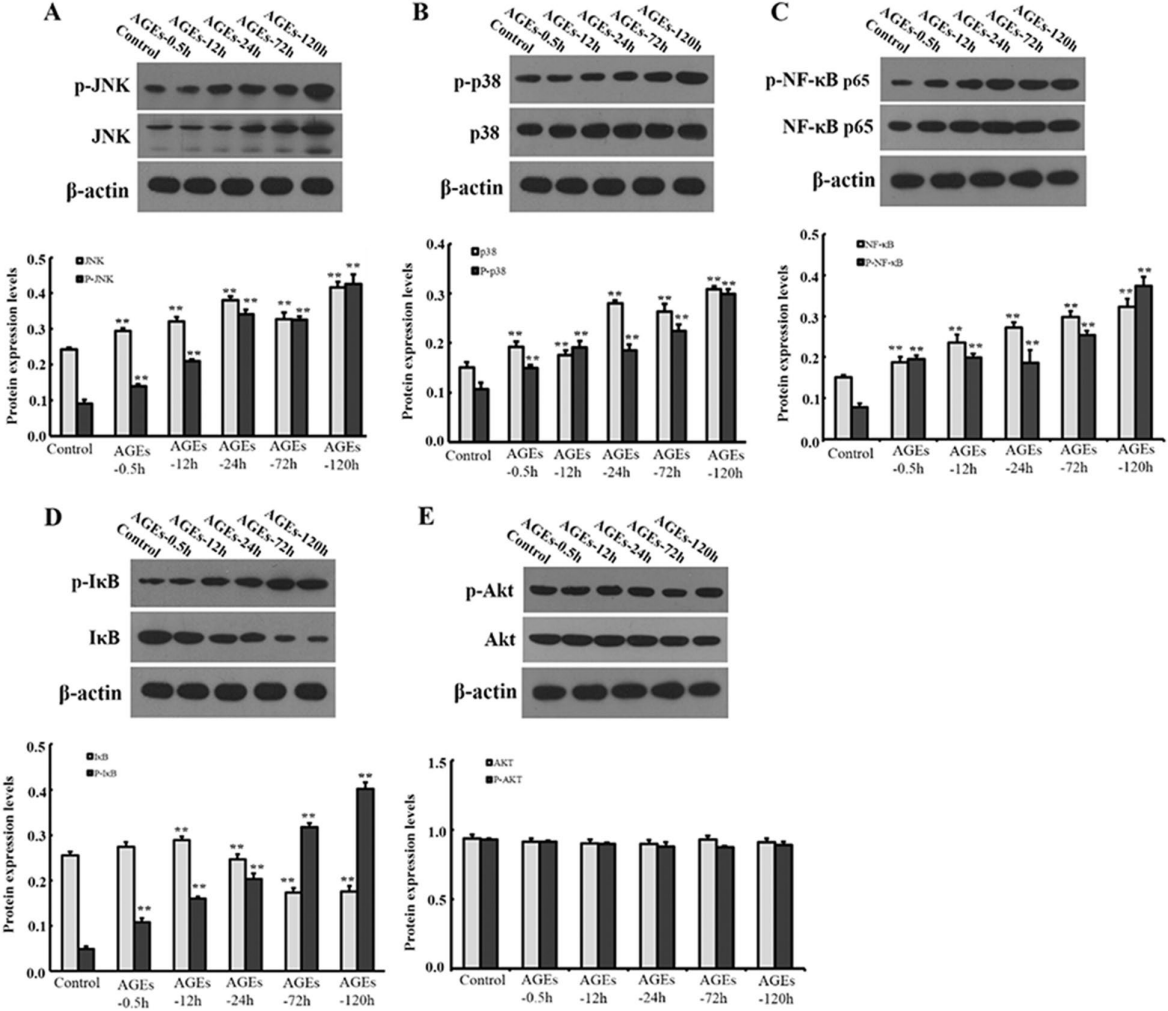
Effect of AGEs on protein expression levels of factors in MAPK signaling pathway. Data are expressed as means ± SD. (*n* = 3). **P < 0.05 vs. control group. **a** 30 min; **b** 12 h; **c** 24 h; **d** 72 h; **e** 120 h

BMSCs were treated with 0.25 mM AGEs for 30 min, 12 h, 1 day, 3 days and 5 days, respectively. The mRNA expression levels are shown in Fig. 3. With the prolongation of the action time, AGEs notably increased the mRNA expression levels of NF-κB p65, JNK and P38, and decreased the mRNA expression levels of IκB (*P* < 0.05). However, there was no significantly difference in Akt mRNA expression level (*P* > 0.05). Moreover, we also detected the protein expression levels of related factors in MAPK signaling pathway (Fig. 4). With the prolongation of action time, AGEs notably increased the protein expression levels of NF-κB p65, p-NF-κB p65, JNK, p-JNK, p-P38, P38 and p-IκB, but decreased the expression of IκB (*P* < 0.05). However, there was no significantly difference in protein expression level of Akt and p-Akt (*P* > 0.05).

### The effect of JNK and P38 signaling pathway in the proliferation of BMSCs induced by AGEs

**Fig. 5:**
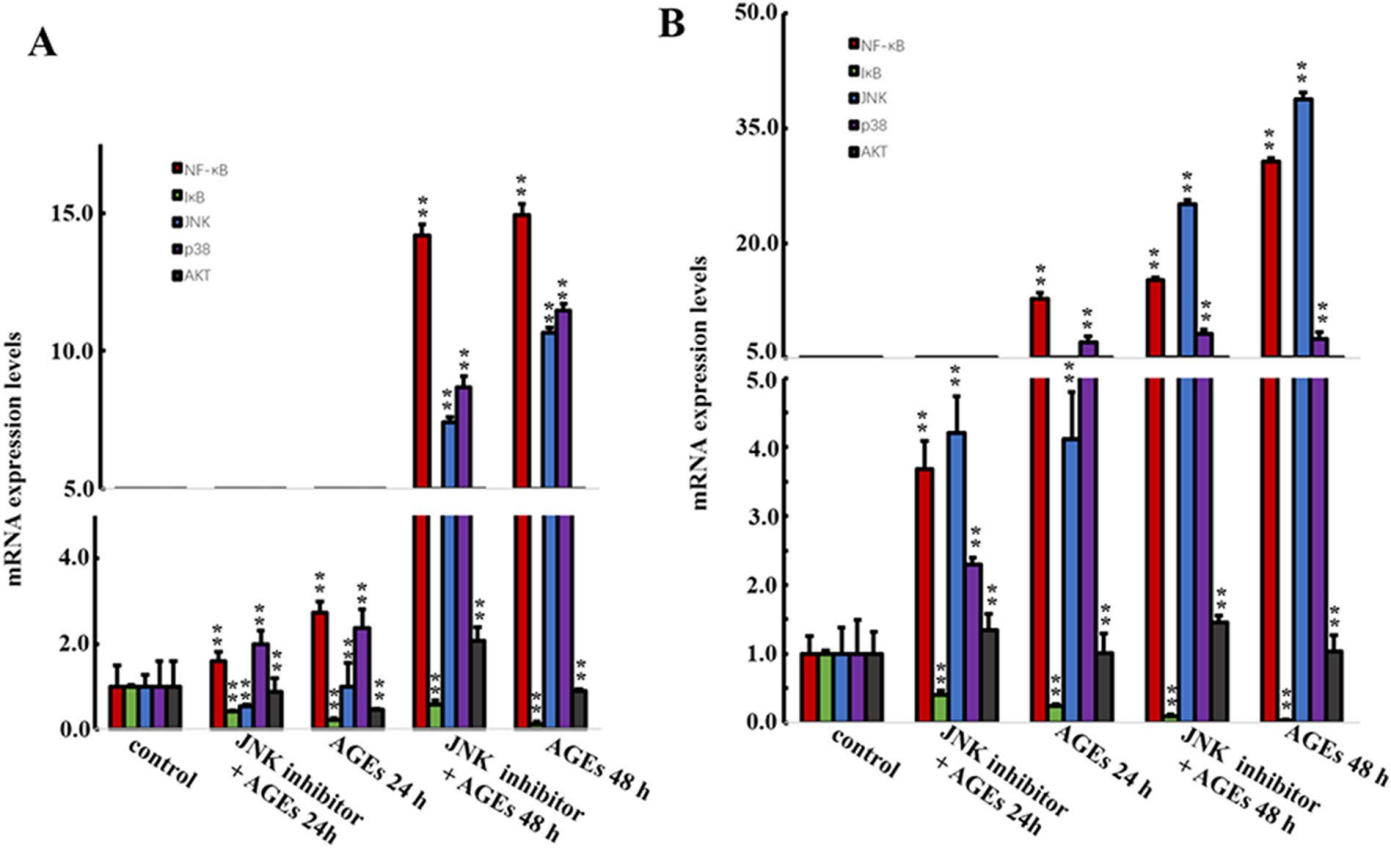
Effect of JNK and P38 inhibitors on AGEs in mRNA expression levels of factors in MAPK signaling pathway. Data are expressed as means ± SD (*n* = 3). **P < 0.05 vs. control group

**Fig. 6:**
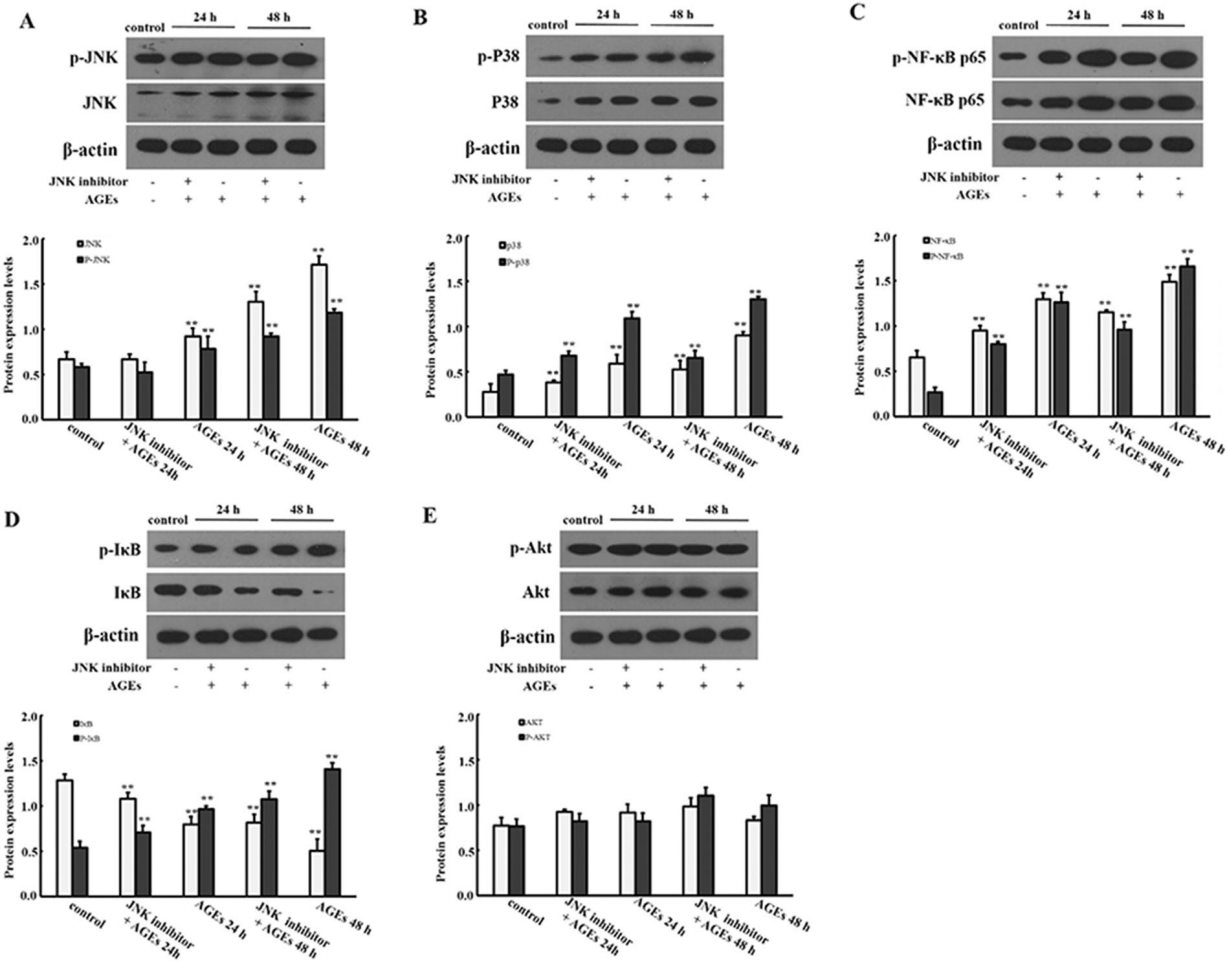
Effect of JNK inhibitor on AGEs in protein expression levels of factors in MAPK signaling pathway. Data are expressed as means ± SD (*n* = 3). **P < 0.001 vs. control group

**Fig. 7:**
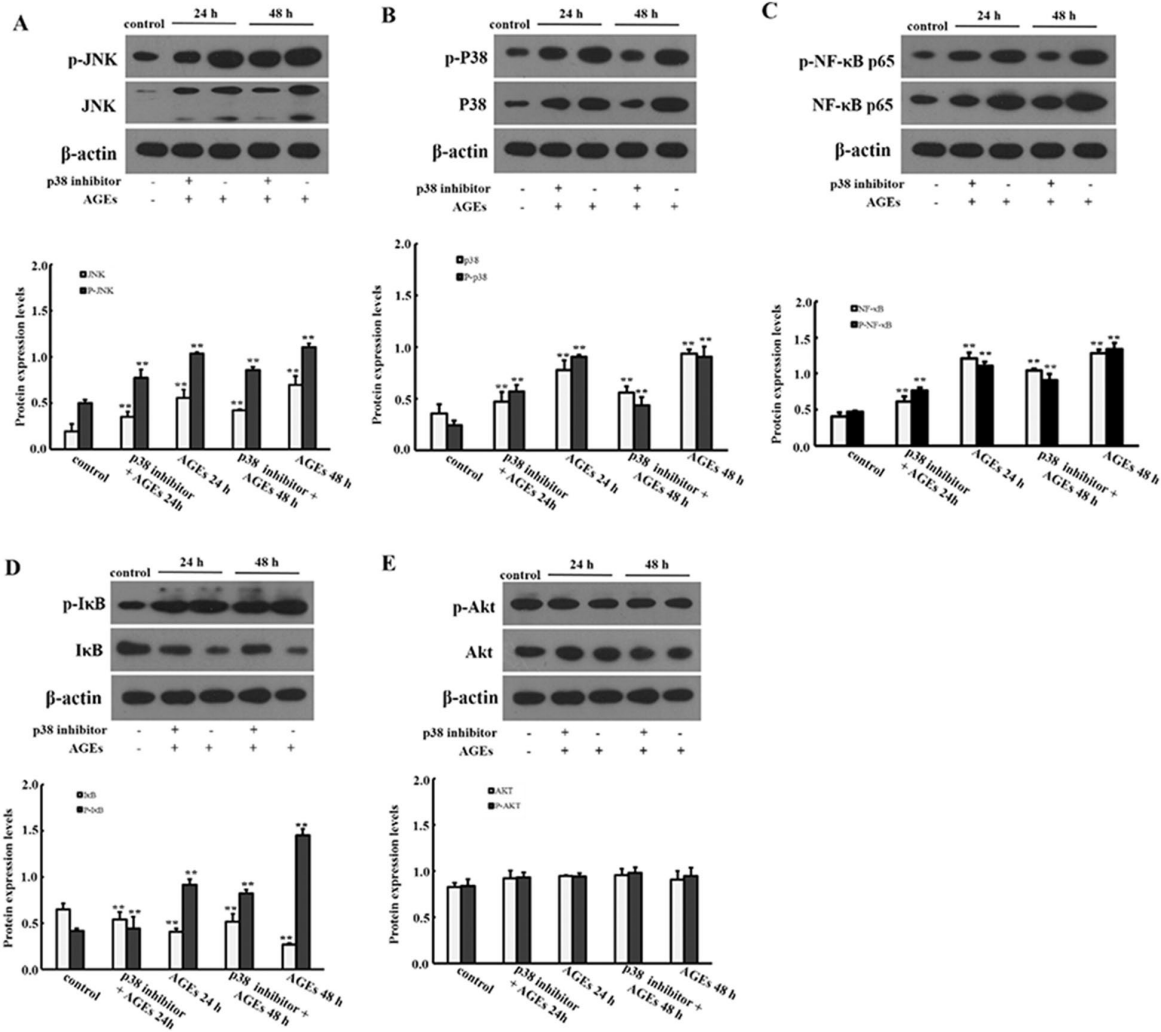
Effect of P38 inhibitor on AGEs in protein expression levels of factors in MAPK signaling pathway. Data are expressed as means ± SD (*n* = 3). **P < 0.001 vs. control group

BMSCs were induced by JNK inhibitor (SP600125) and P38 inhibitor (SB203580) for 30 min, then 0.25 mg/ml AGEs was added to BMSCs for 24 h and 48 h. As shown in Fig. 5, the mRNA expression levels of NF-κB p65, P38 and JNK were significantly decreased when JNK inhibitor and P38 inhibitor were added (*P* < 0.05). However, there was no significantly difference in Akt mRNA expression level when JNK inhibitor and P38 inhibitor were added (*P* > 0.05). In addition, we also evaluated the protein expression levels. As shown in Figs. 6 and 7, the protein expression levels of NF-κB p65, p38, JNK with phosphorylation and non-phosphorylation and IκB phosphorylation in BMSCs were significantly decreased, while IκB non-phosphorylation was significantly increased when JNK inhibitor and P38 inhibitor were added (*P* < 0.05).

## Discussion

AGEs, as glycosylated products of protein and collagen in hyperglycemia, mediate their biological effects by binding to their receptor of RAGE [13]. RAGE is a kind of membrane protein, which can be divided into extracellular domain, transmembrane domain and intracellular segment, and belongs to immunoglobulin superfamily. RAGE is expressed on many cell surfaces, such as monocytes, megaphiles, endothelial cells, smooth muscle cells and fibroblasts. When cells are activated or stressed (hyperglycemia, inflammation), the expression of RAGE is significantly increased. AGEs and RAGE can trigger a variety of signal transduction pathways, such as NADPH oxidase ROS pathway, phosphatidylinositol 3-kinase (PI3-K)—protein kinase B (PKB/Akt) pathway and mitogen-activated protein kinase (MAPK) pathway [14]. It involves extracellular signal regulatory enzymes, including ERK, p38, JNK and other upstream factors, further activating nuclear factor-κB (NF-κB) cascade to play a biological role [15]. The purpose of this study was to detect the effect of AGEs on proliferation of BMSCs, and to analyze the role of PI3K/Akt and MAPK signaling pathway in AGEs–RAGE axis.

CCK-8 test showed that there was a dose and time correlation between AGEs and the activity of BMSCs. With the increase of concentration of AGEs, the cell activity gradually decreased. When the dose exceeded the critical dose of 0.5 mM, the cell activity also decreased significantly with the increase of action time. Therefore, AGEs were selected as the best dosage of 0.25 mM.

NF-κB is a protein family composed of a group of transcription factors with similar structure, and it is an important signaling molecule in intracellular signal transmission. The classical activation mechanism of NF-κB is as follows [16]. In the resting state, NF-κB and its inhibitor IκB exist in the cytoplasm in the form of trimer, and the nuclear localization signal is masked and inactive. After stimulated by the external environment, the trimer dissociates and releases NF-κB, whose nuclear localization signal is exposed, which leads NF-κB into the nucleus and specifically combines with the corresponding target sequence. It starts regulating the transcription of multiple genes, and plays an important role in the immune response and inflammatory response. This study confirms that AGEs (0.25 mM) can promote the activation of NF-κB in BMSCs. The protein expression levels of JNK, p-JNK, p38 and p–p38 in upstream factors were increased, but the protein expression of p-Akt and Akt had no statistical difference, suggesting that JNK and P38 signaling pathways but not PI3K Akt pathway were involved in the effect of AGEs on BMSCs. In addition, during the proliferation of NF-κB, the increased expression of p-IκB protein was accompanied by the decreased expression of IκB. The activation mechanism of NF-κB mediated by AGEs–RAGE was verified. Under the stimulation of AGEs, p-IκB, NF-κB trimer degraded and released NF-κB, which showed that NF-κB pathway was activated and played a biological role.

To verify the hypothesis that JNK and P38 signal pathways are involved in AGEs regulation, JNK and p38 signal pathway inhibitors were added, respectively. The results showed that AGEs had a significant effect on BMSCs with stem cell properties and multi-directional differentiation potential. After blocking the pathway, the originally increased mRNA and protein levels of NF-κB p65, p38, JNK were decreased in BMSCs induced by AGEs. In addition, the increased protein levels of p-NF-κB p65, p-p38, p-JNK and p-P38 were also decreased. It is concluded that JNK and p38 signaling pathways play a synergistic role in the differentiation of BMSCs by AGEs.

There are some disadvantages in present study. First, there is no evidence in vivo about the effect and mechanism of AGEs on BMSCs. Furthermore, only CCK-8 method is used to detect cell proliferation, and there is no more method to detect cell proliferation.

## Conclusion

In conclusion, in this study, BMSCs closely related to alveolar bone resorption in periodontal diseases were studied. It was confirmed that AGEs could inhibit BMSCs proliferation and NF-κB expression. This process was affected by JNK and p38 signal regulation in MAPK pathway, and had nothing to do with PI3K/Akt signal pathway. In the future, we can further analyze the effects of AGEs–RAGE axis on the biological effects of BMSCs, such as the detection of some active cytokines and the expression of some osteogenic genes. In addition, to fully understand the effect of hyperglycemia on periodontal soft and hard tissues, we can continue to explore the effect of AGEs on monocytes, osteoblasts, osteoclasts and potential interaction of cells.

## Data Availability

Not applicable.

## Abbreviations

AGEs: Advanced glycosylation end products
BMSCs: Bone-marrow stromal cells
CCK-8: Cell counting kit-8
EFP: European Periodontal Association
AAP: American Periodontal Association
ROS: Reactive oxygen species
BSA: Bovine serum albumin
PMSF: Phenylmethylsulfonyl Fluoride
EDTA: Ethylene diamine tetraacetic acid
FBS: Fetal bovine serum
DMEM: Dulbecco’s modified eagle medium
SD: Sprague–Dawley
RBC: Red blood cell
OD: Optical density
SD: Standard deviation
ANOVA: Analysis of variance
GLM: General linear model
PI3-K: Phosphatidylinositol 3-kinase
PKB/Akt: Protein kinase B
pathway MAPK: Mitogen-activated protein kinase
NF-κB: Nuclear factor-κB

## References

[1] Petersen PE, Ogawa H (2012). The global burden of periodontal disease: towards integration with chronic disease prevention and control. Periodontol.

[2] Papapanou PN (1996). Periodontal disease: epidemiology. Ann Periodontol.

[3] Khader YS, Dauod AS, El-Qaderi SS, Alkafajei A, Batayha WQ (2006). Periodontal status of diabetics compared with nondiabetics: a meta-analysis. J Diabetes Complications.

[4] Li Z, Sha YQ, Zhang BX, Zhu L, Kang J (2011). The effect of community periodontal health care intervention on periodontal health and blood glucose metabolism in patients with type 2 diabetes. J Peking Univ.

[5] Matthews DC (2002). The relationship between diabetes and periodontal disease. J Can Dent Assoc.

[6] Taylor JJ, Preshaw PM, Lalla E (2013). A review of the evidence for pathogenic mechanisms that may link periodontitis and diabetes. J Clin Periodontol.

[7] Volpe CMO, Villar-Delfino PH, Dos Anjos PMF, Nogueira-Machado JA (2018). Cellular death, reactive oxygen species (ROS) and diabetic complications. Cell Death Dis.

[8] Russo I, Frangogiannis NG (2016). Diabetes-associated cardiac fibrosis: cellular effectors, molecular mechanisms and therapeutic opportunities. J Mol Cell Cardiol.

[9] Elenkova M, Tipton DA, Karydis A, Stein SH (2019). Vitamin D attenuates human gingival fibroblast inflammatory cytokine production following advanced glycation end product interaction with receptors for AGE. J Periodontal Res.

[10] Taiyeb-Ali TB, Cheta Raman RP, Vaithilingam RD (2000). Relationship between periodontal disease and diabetes mellitus: An Asian perspective. Periodontol.

[11] Jiang N, Guo W, Chen M, Zheng Y, Zhou J, Kim SG (2016). Periodontal ligament and alveolar bone in health and adaptation: tooth movement. Front Oral Biol.

[12] Shen Y, Guo S, Chen G, Ding Y, Wu Y, Tian W (2019). Hyperglycemia induces osteoclastogenesis and bone destruction through the activation of Ca2+/calmodulin-dependent protein kinase II. Calcif Tissue Int.

[13] Sahajpal NS, Goel RK, Chaubey A, Aurora R, Jain SK (2019). Pathological perturbations in diabetic retinopathy: hyperglycemia, AGEs, oxidative stress and inflammatory pathways. Curr Protein Pept Sci.

[14] Chaves MM, Costa DC, de Oliveira BF, Rocha MI, Nogueira-Machado JA (2009). Role PKA and p38 MAPK on ROS production in neutrophil age-related: lack of IL-10 effect in older subjects. Mech Ageing Dev.

[15] Southerlan JH, Taylor GW, Moss K, Beck JD, Offenbacher S (2000). Commonality in chronic inflammatory diseases: periodontitis, disbetes, and coronary artery disease. Periodontol.

[16] Bierhaus A, Schiekofer S, Schwaninger M, Andrassy M, Humpert PM, Chen J (2001). Diabetes-associated sustained activation of the teanscription factor nuclearfactor-kappaB. Diabetes.

